# Unique Aspects of Cryptochrome in Chronobiology and Metabolism, Pancreatic *β*-Cell Dysfunction, and Regeneration: Research into Cysteine414-Alanine Mutant CRY1

**DOI:** 10.1155/2016/3459246

**Published:** 2016-12-26

**Authors:** Satoshi Okano

**Affiliations:** Research Center for Molecular Genetics, Institute for Promotion of Medical Science Research, Yamagata University Faculty of Medicine, Yamagata 990-9585, Japan

## Abstract

Cryptochrome proteins (CRYs), which can bind noncovalently to cofactor (chromophore) flavin adenine dinucleotide (FAD), occur widely among organisms. CRYs play indispensable roles in the generation of circadian rhythm in mammals. Transgenic mice (Tg mice), ubiquitously expressing mouse CRY1 having a mutation in which cysteine414 (the zinc-binding site of CRY1) being replaced with alanine, display unique phenotypes in their circadian rhythms. Moreover, male Tg mice exhibit symptoms of diabetes characterized by beta-cell dysfunction, resembling human maturity onset diabetes of the young (MODY). The lowered proliferation of *β*-cells is a primary cause of age-dependent *β*-cell loss. Furthermore, unusually enlarged duct-like structures developed prominently in the Tg mice pancreases. The duct-like structures contained insulin-positive cells, suggesting neogenesis of *β*-cells in the Tg mice. This review, based mainly on the author's investigation of the unique features of Tg mice, presents reported results and recent findings related to molecular processes associated with mammalian cryptochromes, especially their involvement in the regulation of metabolism. New information is described with emphasis on the aspects of islet architecture, pancreatic *β*-cell dysfunction, and regeneration.

## 1. Various Functions of Cryptochromes in Animals

**Figure 1:**
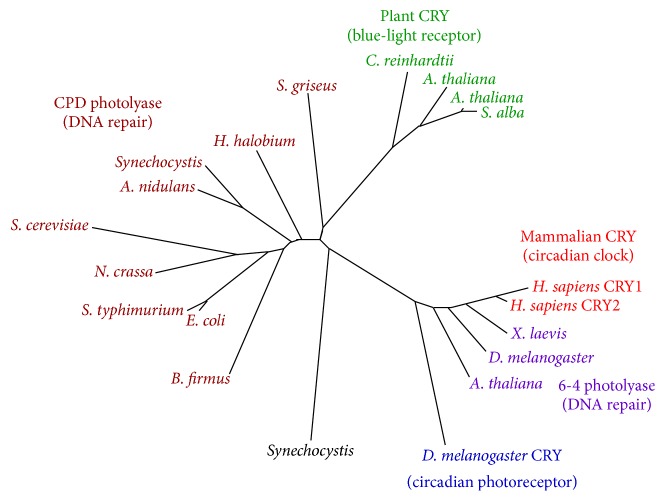
Phylogenetic tree of photolyase/cryptochrome family proteins (adapted from [2]).

Before addressing the main subject, one can concisely outline the diverse roles of cryptochromes in animals along with the track of related research. Cryptochrome proteins, which can bind noncovalently to cofactor (chromophore) flavin adenine dinucleotide (FAD), occur widely among organisms [1]. The term cryptochrome itself was coined around 1970 to describe hitherto unknown photoreceptors in plants:* crypto* means hidden or mysterious in Greek. The cryptochromes are evolutionally conserved and are structurally related to photolyase, a DNA repair enzyme [1].  Figure 1 portrays a phylogenetic tree of photolyase/cryptochrome family proteins referred to in the literature [2]. Efforts undertaken for more than half century to elucidate the molecular processes of DNA repair began with the discovery of photolyase and culminated in the awarding of a Nobel Prize in Chemistry in 2015 [3]. Cryptochromes also play fundamentally important roles in the circadian clock of organisms living on earth, enabling them to adapt to daily 24 h cycles.

Mammals exhibit various physiological and behavioral processes that show rhythms: sleep/wake activity, secretion of hormones, and metabolism fluctuate rhythmically in approximately 24 hr cycles. These phenomena are regulated by an internal circadian clock in the body of each individual [4]. Already by the 1970s, the central site of the mammalian circadian clock was singled out anatomically as the suprachiasmatic nucleus (SCN) [5–7], but the molecular clockwork in the SCN remained elusive until the last few years of the 20th century [4]. Along with the advance of molecular biology and genetics, the long sought-after molecular mechanism of the mammalian circadian rhythm was discovered during a short period in the late 1990s [4]. The clock genes were cloned during that period [4]. Mammalian cryptochromes 1 and 2 (*Cry1 *and* Cry2*) were also identified and characterized during that time [8–11]. A series of knockout (KO) studies in mice played crucial roles in determining the function of CRYs in mammals: the Yasui group produced mutants that were defective in each of* Cry1* or* Cry2* or in both genes. This research group, in conjunction with Van Der Horst et al., found that the mutants exhibited no rhythmic behavior when kept in constant darkness [12]. Independently, using a similar double knockout strategy, another group reached fundamentally identical conclusions [13]. The result, along with those of an in vitro study [14], proved that CRYs are indispensable components of clock proteins. Single* Cry1* KO mice show a shorter period phenotype but single* Cry2* KO mice present a longer period phenotype than that of wild-type control mice [12, 13]. These results suggest that the functions played by these CRYs in the regulation of circadian clock are not the same.

**Figure 2:**
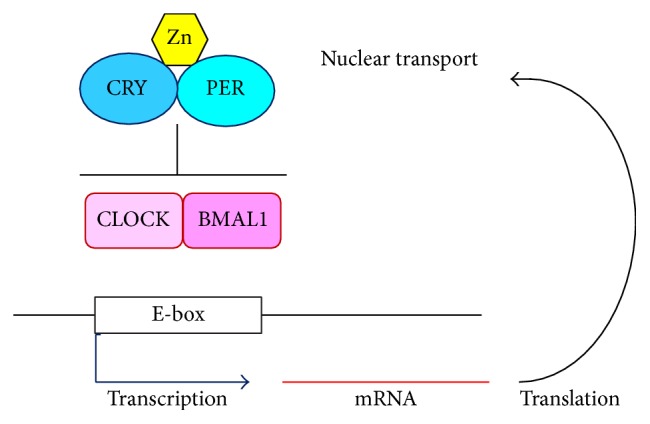
Molecular transcriptional/translational feedback loop (TTFL) model for mammalian circadian clock.

By 2000, the basic molecular mechanism of mammalian circadian rhythm was identified as a cyclic transcriptional/translational feedback loop (TTFL), which comprises four classes of proteins: Periods (PERs), cryptochromes (CRYs), CLOCK, and BMAL1 [4]. A schematic diagram of mammalian TTFL is presented in Figure 2. In the feedback loop, CRY proteins form dimers with PER proteins in the cytoplasm. Thereafter, they enter the nucleus and play the role of a transcriptional repressor to the CLOCK/BMAL1 complex [4] (Figure 2). Nuclear receptor family proteins of two kinds, REV-ERBs and RORs, form another loop of the molecular clock [4, 16]. The second molecular loop plays a role in stabilizing TTFL [16]. Regarding* Drosophila melanogaster*, the* Drosophila* Period gene was cloned originally long before in 1984, but the molecular mechanism of the circadian rhythm was elucidated only during the last few years of the 20th century, almost simultaneously with the mammalian circadian rhythm [17]. A negative feedback loop similar to that of TTFL in mammals was identified comprising four proteins: Period, Timeless, CLOCK, and Cycle [17]. The main role of* Drosophila *CRY turned out to be that of a photoreceptor [1, 17, 18]. Still another aspect of cryptochromes that is recently attracting many researchers' interest is the function of “magnetoreception.” For instance, coherent quantum mechanical analyses [19] predicted that photoexcitation of cryptochrome proteins in the avian retina generated radical pairs (pairs of electron spins), which enable migratory birds to detect the direction of earth's weak magnetic field. Results of that study suggest that the radical pair magnetoreception in cryptochrome can be regarded as a quantum biology phenomenon [19].

Mammalian CRYs are subject to various modifications posttranslationally, as described below. Such multilateral regulations of CRYs are believed to regulate or fine-tune the oscillation of mammalian molecular clocks. Degradation of CRY proteins is conducted via the ubiquitin system. AMP-activated protein kinase (AMPK), which is known to work as a metabolic sensor, phosphorylates CRY proteins [20] and regulates CRY protein stability by leading CRY proteins to bind to a ubiquitin ligase: FBXL3 [21–23]. FBXL21, another ubiquitin ligase, stabilizes CRY proteins [24, 25]. Ubiquitin-specific protease 7 (USP7) deubiquitinates CRYs and thereby stabilizes CRYs [26]. Furthermore, posttranscriptional regulation has been reported to occur by hnRNP Q, an RNA-binding protein, which controls translation of* Cry1* mRNA [27].

## 2. Structural-Biological Analyses of CRYs

Recent X-ray crystallographic analysis conducted along with investigations of molecular biology has provided important clues that have elucidated the molecular roles of CRYs. Structural studies have elicited details of interaction of CRYs with FBXL3 and with PER2, as described below. For regulating circadian periods and/or metabolism by the ability to modulate CRY molecular structures, various chemicals have been created which bind to CRYs and which affect them. One such chemical is KL001 [28]. Reportedly, FBXL3 binds to CRY2 by occupying CRY2's binding pocket to FAD [29]. Also, KL001 inhibits FBXL3-dependent degradation of CRYs by competing with FAD for CRY binding, causing lengthening of the period of the molecular clock [28, 30]. In terms of metabolism, the role of CRYs in the regulation of hepatic gluconeogenesis has been demonstrated. In addition to the effect on the circadian period, in primary hepatocytes, KL001 affects gluconeogenesis through action to CRYs [28]. CRY inhibits glucagon-stimulated cAMP production through regulating a Gs *α* subunit of a G protein [31]. SREBP1c regulates gluconeogenesis by CRY1-mediated FOXO1 degradation [32]. CRYs also regulate NF*κ*B signalling by binding directly to adenylate cyclase and by limiting the production of cAMP [33]. CRYs bind directly to the glucocorticoid receptor and change the transcriptional response to glucocorticoids [34, 35]. The crystal structure of a CRY1-PER2 complex has been demonstrated by Schmalen et al. [36]. They presented new aspects of the molecular regulation of CRYs: zinc ion plays important roles in the formation of the CRY1-PER2 protein complex [36]. Also, cysteine414, which functions as an intermolecular zinc-binding site located in CRY1, plays a pivotal role in the binding of CRY1 to PER2, in conjunction with three other zinc-binding amino acid residues in both CRY1 and PER2 [36]. Another group determined the crystal structure of CRY2-PER2 complex [37] and showed that the relevant cysteine in CRY2 [38] also functions as an intermolecular zinc-binding site [37]. Details of the structure of CRYs based on recent structural analyses are discussed in a review [39].

## 3. Unusual Circadian Rhythms and Diabetes Mellitus in C414A CRY1 Tg Mice

The author previously generated transgenic mice ubiquitously overexpressing mouse CRY1 as well as mouse CRY1 having a mutation, cysteine414 being replaced with alanine (C414A CRY1 Tg mice: previously designated as CRY1-AP Tg mice) [38]. Actually, C414A CRY1 severely reduces its binding ability to PER2 by in vitro examination [36]. The author generated two expression lines for C414A CRY1 Tg mice: high (H) and low (L) lines [38]. Fundamentally, they show a similar phenotype in terms of pathophysiological phenotypes [40]. Mice overexpressing intact CRY1 (CRY1 Tg mice) generated almost normal circadian rhythms in wheel-running activity [38]. In sharp contrast, the mice overexpressing the mutant CRY1 (hereinafter designated simply as Tg mice) displayed long free-running periods depending on the expression levels of transgenes [38]. The following refers only to the H line of Tg mice. They also showed abnormalities in both light-entrained [38] and food-entrained locomotor rhythms [15]. Detailed unique features of the SCN, which governs such unusual rhythms in Tg mice, are discussed in the literature [15]. In the Tg mice liver, the circadian expression of E-box-driven genes,* Per2* and* Dbp*, was depressed markedly [38], suggesting the hyperrepressive ability in C414A CRY1 to the E-box-driven clock gene and also to E-box-driven clock-controlled genes in vivo. Results demonstrated that* Cry*-deficient MEF cells show that C414A CRY1 actually depresses the amplitude of E-box-driven molecular clock and lengthens its period [37], which is inconsistent with the feature of Tg mice in terms of their extreme long free-running periods [38].

The author found that Tg mice show diabetes mellitus [38] in addition to unusual circadian behaviors [15, 38]. The features of diabetes are summarized as follows [40]. (1) Symptoms in Tg mice show sex-dependence: only male Tg mice show diabetes. (2) Tg mice at six weeks, the age at which blood glucose levels begin to elevate, already show glucose intolerance and exhibit no discernible insulin resistance. (3) At 9 weeks of age, neither hypercholesterolemia nor hypertriglyceridemia was observed in Tg mice. (4) Immunohistochemical analyses conducted at 19 and 40 weeks demonstrated that the insulin-stained area in the islet of Tg mice was smaller than that of wild-type controls. The extent of the decrease progressed age-dependently. (5) Levels of glucose-stimulated insulin secretion in Tg mice at 27 weeks were reduced compared with those of wild-type controls. Results suggest that, in Tg mice, the function of the insulin secretion of *β*-cell and the development of *β*-cell from a young age must be impaired to a certain degree. Consequently, Tg mice were expected to serve as an animal model of the insulin-secretory defect in human, such as maturity onset diabetes of the young (MODY [41]), a disease that is characterized by early onset and *β*-cell dysfunction [40]. At 40 weeks, male Tg mice showed higher serum triglyceride concentrations than wild-type controls did [38]. This phenomenon is attributable to the secondary effects of severe diabetes (diabetic lipemia) and does not contradict previously reported conclusions [40]. Actually, the blood glucose level increases age-dependently and reaches an extremely high level in male Tg mice [38, 40]. Already at 40 weeks of age (ca. 10 months of age), Tg mice exhibit various severe symptoms of diabetes: a decrease in body weight accompanied by swelling out of lower abdomen and wet hindquarters caused by excessive polydipsia and polyuria [38, and unpublished data]. The author and colleagues have reported that lowered proliferation of *β*-cells is responsible for age-dependent *β*-cell loss in Tg mice [42]. The expression of transcription factors MAF-A and PDX-1, which are known to be important for the *β*-cell maturation and function, is reduced in the islet of Tg mice as they age [42]. The decreased expression of* Insulin*,* Glucokinase*, and* Glut2* from a young age in the pancreas of Tg mice has also been reported [42]. In addition to the findings on C414A CRY1 Tg mice, the results of many intensive studies have implicated the involvement of clock genes in *β*-cell function [43–50]. The physiological consequences of various clock gene-modified animals are described in a review [51].

## 4. Characteristic Features of Gene Expressions in the Islets of Young Tg Mice

Recent DNA microarray analysis of islets derived from Tg mice at 4 weeks of age showed that the mRNA levels of E-box-driven clock genes were markedly reduced in the islets of Tg mice (Table 1) [52], indicating malfunction of the molecular clock in the islets of Tg mice. Clock-related and metabolism-related transcription factors, DBP, RER-ERB*α*, and ROR*γ*, were reduced severely in the pancreas or islets of Tg mice [42, 52, 53]. Reportedly, these proteins are expressed in pancreatic *β*-cells. They can directly or indirectly affect the function of *β*-cells [46, 54–56]. In line with the decreased proliferation of *β*-cells [42], the mRNA levels of various genes involved in cell-cycle control were altered [52]. Moreover, the expressions of various secretory proteins including inflammatory cytokines, chemokines, growth factors, and tissue remodelling factors were promoted in the islet of Tg mice [52]. This expression pattern is reminiscent of that of senescence-associated secretory phenotype (SASP) [57]. In silico analyses were conducted to trace the causes of this peculiar pattern of expression in the islet, based on DNA microarray data [53]. Results show that the overexpression of C414A CRY1 influences various signalling pathways in the islet. Particularly, aberrant activation by C414A CRY1 in the NF*κ*B-mediated and also in the glucocorticoid receptor- (GR-) mediated signalling pathways can play central roles in unusual features of *β*-cells in Tg mice [53]. In harmony with that, NF*κ*B signalling is known to play important roles in the induction of SASP [57]. The involvement of CRYs in both GR-mediated and NF*κ*B-mediated signalling pathways has already been reported as described above [33–35]. Glucocorticoids influence *β*-cell function and also their population [58, 59]. It is noteworthy that the mRNA and protein levels of RGS (regulator of G protein signalling) 4 increased markedly [53]. By inactivating G*α*q, a subtype of G protein *α* subunits, it acts as a negative regulator of insulin release from pancreatic *β*-cells via M_3_ muscarinic acetylcholine receptor [60]. Disturbance in G*α*q signalling pathways can affect not only insulin secretion but also the proliferation of *β*-cells in mice [61].* Rgs16* is an E-box-driven clock gene [62]. RGS16 is a protein known to inhibit G*α*i/o [62]. Actually, the mRNA level of the* Rgs16* was reduced in the islet of Tg mice [53], which is consistent with its reduced expression in the liver of Tg mice [15]. A recent report described that RGS16 regulates not only insulin secretion but also *β*-cell proliferation [63]. Consequently, disturbance in the signalling pathways mediated by RGS4 and RGS16 can cause not only insulin secretion but also the proliferation of *β*-cells in Tg mice. Collectively, in addition to the dysfunction of circadian clock in the islet, the combined influences of multiple pathways can cause the insulin-secretory defect and can cause severely decreased proliferation of *β*-cells in Tg mice from a young stage.

## 5. Abnormality in the Islet Architecture of Tg Mice

**Figure 3:**
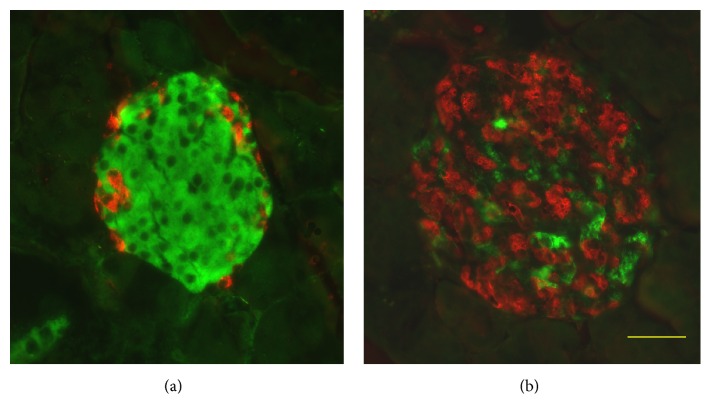
Coimmunostaining for insulin and glucagon of islets. Pancreas sections from wild-type (a) and Tg (b) mice at 40 weeks of age costained with antibodies to insulin (green) and glucagon (red). Bar, 40 *μ*m.

In Tg mice, the increase of the *α*-cell/*β*-cell ratio and gross abnormality in the distribution of *α*-cells becomes conspicuous as they grow (Figure 3) [42]. The islet area per pancreas in Tg was smaller than that in wild-type controls [42]. To investigate the related mechanisms, *α*-cell proliferation was measured using immunohistochemical analysis at 2 weeks of age. Results showed no significant difference in *α*-cell proliferation between Tg and wild-type mice [53]. In addition, the glucagon-positive area per islet was increased from a young stage at 2 weeks [53] and 4 weeks [42]. Consequently, the unusual composition of cells is attributable in the literature mainly to “collapse of islets” [64] because of the specific loss of *β*-cells that mainly results from decreased proliferation in Tg mice.

## 6. Unusual Ductal Structures in the Pancreas

**Figure 4:**
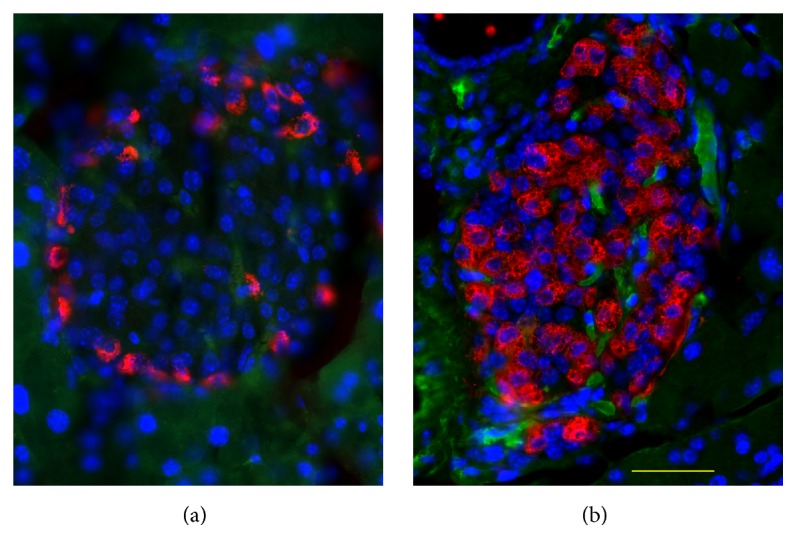
Increase in vascular formation in the islets of Tg mice. Pancreas sections from wild-type (a) and Tg (b) mice at 40 weeks of age costained with antibodies to PECAM1 (green) and glucagon (red) and counterstained with DAPI (blue) for nuclear staining. Bar, 40 *μ*m.

**Figure 5:**
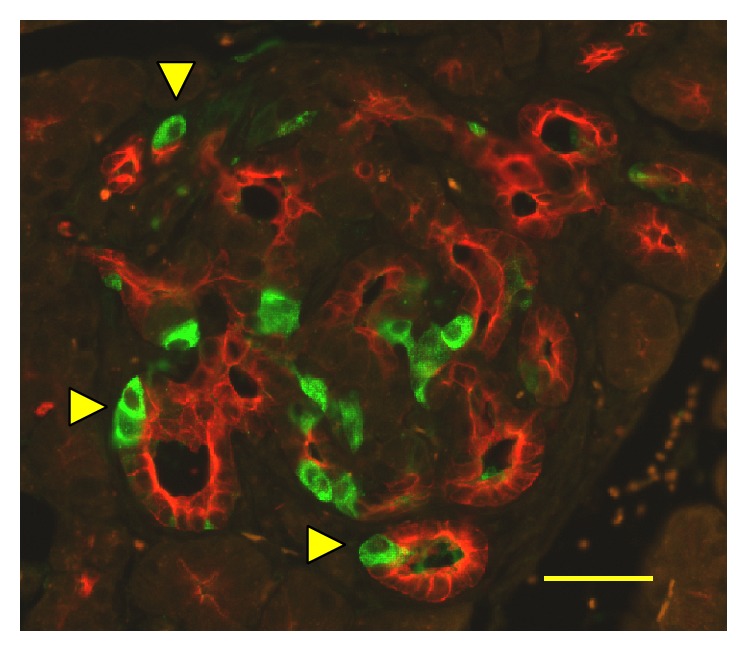
Insulin-expressing cells in the Tg mice ductal structures. Pancreas sections from Tg mice at 40 weeks of age costained with antibodies to insulin (green) and cytokeratin 17/19 (red). Yellow triangles denote insulin-positive cells in the structure. Bar, 40 *μ*m.

**Figure 6:**
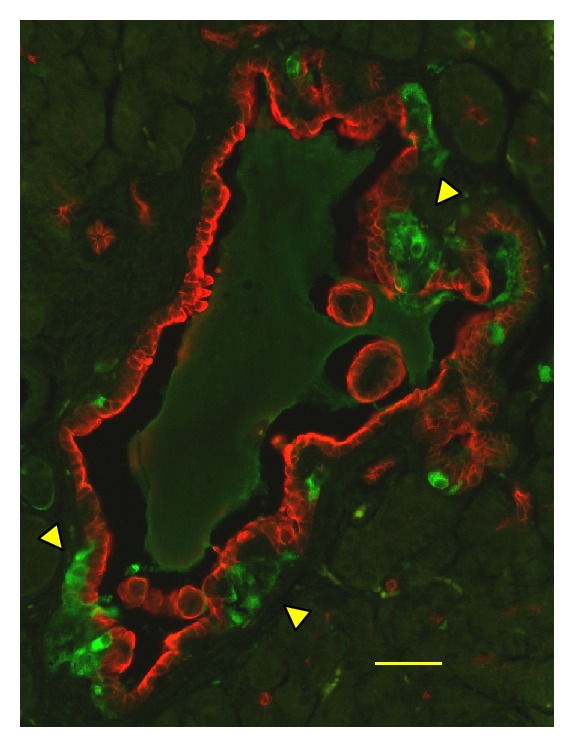
Clusters of insulin-positive cells located near the duct-like structure in Tg mice. Pancreas sections from Tg mice at 40 weeks of age were costained with antibodies to insulin (green) and cytokeratin 17/19 (red). Bar, 40 *μ*m.

Immunohistochemical analyses have shown that unusually enlarged duct-like SOX9 positive structures (SOX9 is a transcription factor known as a ductal marker as well as a pancreatic endocrine progenitor marker) developed more conspicuously in pancreases in Tg mice than in those of wild-type controls [52, 53]. In Tg mice, this phenomenon became discernible histologically at the mature stage [52, 53]. Numerous duct-like cells were PCNA-positive [53], suggesting that enhanced proliferation of the cells is involved in the development of the duct-like structures. At the mature stage, higher numbers of the vascular networks in the islets in Tg mice than those of wild-type mice were observed (Figure 4) [53]. Consistent with that observation, from the young stage, the mRNA level of* Angiopoietin 2* was elevated in Tg mice islets [52]. It is noteworthy that previous reports have described that transgenic mice of the constitutively active form of constituents of insulin signalling pathway (*Akt* [65],* Foxo1* [66]) showed similar expanded ductal structures, which contain proliferation marker-positive cells. Particularly, transgenic mice in which the overexpression of constitutively active form of FOXO1 in the pancreas uses* Pdx-1* promoter showed a similar phenotype to that of C414A CRY1 Tg mice: The FOXO1 Tg mice showed impaired glucose tolerance with the reduction in the *β*-cell mass accompanied by the reduction of MAF-A and PDX-1 [66]. In addition, the formation of hypervascular networks was observed in the islets of the FOXO1 Tg mice [66]. In this respect, a recent report describing that CRY1 is involved in the degradation of nuclear FOXO1 in conjunction with a ubiquitin ligase, MDM2 [32], is quite suggestive of the mechanism of unusual ductal structure growth. Possibly, C414A CRY1 inhibits dominant-negatively normal CRY1 for the degradation of FOXO1. Thereby, it functions as an activator of FOXO1 mediated signalling pathway in the Tg mice pancreas. Therefore, the overactivation of FOXO1 might be the primary cause of the induction of ductal structures in Tg mice. Moreover, deregulation of signalling cascades, such as* Hedgehog*-,* Notch*-, and* Wnt*-signalling pathways, might cause the development of the unusual ductal structures in Tg mice. C414A CRY1 might affect mouse Timeless (TIM; TIM is known as a CRY-binding protein [39]) and might perturb the function of TIM in the pancreas, possibly leading to aberrant activation of the signalling pathways, as described in one report [67]. Actually, some constituents of* Hedgehog*-signalling and* Wnt*-signalling pathways were elevated in the Tg mice islet [52]. Recently, it was proposed that TIM plays a role in DNA damage response in conjunction with PARP1 [68]. Perturbation of TIM by C414A CRY1 might cause the accumulation of DNA damage, which can contribute to the lowered proliferation of *β*-cells in Tg mice. The duct-like structures contained insulin-positive cells (Figure 5) [53]. In addition, the clusters of insulin-positive cells were located near the duct-like structure (Figure 6) [53]. These results strongly suggest that the neogenesis of *β*-cells occurs there. One review has summarized experimentally derived models of *β*-cell neogenesis reported to date and the roles of ductal cells in respect of the source of *β*-cells [69]. At the mature stage, infiltrated macrophage cells were observed frequently in the islet of Tg mice [53]. Chemokine/cytokine-induced epithelial-mesenchymal transition (EMT) in the process of *β*-cell neogenesis from ductal cells was proposed recently [70]. A similar induction mechanism to that of EMT is likely to be involved, at least partially, in generating new *β*-cells from duct-like structures, in which the secreted cytokines and chemokines play relevant roles in Tg mice because of SASP-like characters of *β*-cells [52] and by recruited macrophage cells to the islet [53]. Taken together, remodelling in the vascular network in the islet progresses with age. Infiltrated macrophages might be involved, at least partially, in the processes.

## 7. Future Directions

Regarding cross-talk between the circadian clock and the regulation of functions of *β*-cells, the elucidation of the circadian-hormonal effects is expected to become increasingly important. This trend is exemplified by recent reports: melatonin, a hormone that exhibits some relation to circadian rhythm [71], directly regulates insulin secretion of *β*-cells [72]. Glucocorticoids, the concentrations of which also fluctuate in a circadian manner [71], influence *β*-cell function as discussed above. Moreover, it has been demonstrated that thyroid hormone T_3_ stimulates *β*-cell maturation by increasing MAF-A expression [73]. Circadian control of thyroid hormones is described in a review [74]. Consequently, appropriate timing of the daily exposure of these hormones to *β*-cells is expected to be important not only for the fine-tuning of *β*-cell functions, but also for the maintenance of *β*-cells.

The gross abnormality displayed remarkably in the islet and pancreas in Tg mice demonstrates that CRY functions in mammals are limited not only to the part of TTFL of circadian clock; they also strongly affect *β*-cell proliferation and physiology. Particularly, it is intriguing to imagine that the Zn-binding of CRY-PER complex might have some additional uncovered roles in the *β*-cell function because contents and cellular levels of zinc in *β*-cells are known to be exceptionally high compared with those of other cells [75]. To enhance our understanding of why ubiquitous overexpression of C414A CRY1 especially affects *β*-cells in mice, further experiments must be conducted.

## Figures and Tables

**Table 1:** Expression change in clock-related genes of isolated islets. The mice were kept in LD 14:10 as described [15]. The lighting period was 07:00–21:00, and islets were harvested during 11:00–13:00.

Gene name	Fold change (Tg/wild-type)	
Hif	Down	9.60
Per3	Down	9.48
Ror*γ*	Down	8.68
Bmal1	Up	5.90
Cry1 (endogenous)	Down	4.98
Dbp	Down	4.60
Fbxl21	Down	4.55
Tef	Down	4.52
Per2	Down	3.57
Per1	Down	2.47
Cry2	Down	2.33
